# Commentary: BAFF blockade attenuates DSS-induced chronic colitis *via* inhibiting NLRP3 inflammasome and NF-кB activation

**DOI:** 10.3389/fimmu.2024.1528731

**Published:** 2024-12-24

**Authors:** Jie Guo, Chen-guang Li

**Affiliations:** ^1^ School of Medicine, Shenzhen University, Shenzhen, China; ^2^ Pain Department of Shenzhen Nanshan People's Hospital, Shenzhen, China

**Keywords:** RAW264.7 cells, ASC, expression, NLRP3 inflammasome, LPS

## 1 Introduction

With a discerning and inquisitive interest, we read the paper “BAFF Blockade Attenuates DSS-Induced Chronic Colitis via Inhibiting NLRP3 Inflammasome and NF-κB Activation” published in Frontiers in Immunology (1). In this study, Zhang et al. present compelling evidence for the pivotal role of BAFF (B cell activating factor) in inflammatory bowel disease (IBD). Their findings suggest that BAFF neutralization ameliorates colitis by mitigating inflammation and suppressing NF-κB and NLRP3-related signaling pathways, thus offering a promising therapeutic target for IBD treatment. The author’s present provides valuable insights into the molecular mechanisms underlying IBD pathogenesis and highlights the potential of BAFF blockade as a novel therapeutic approach. In general, this is an excellent piece of research. However, there are a few points in the paper that require further discussion and critical examination.

## 2 Results and discussion

**Figure 8:**
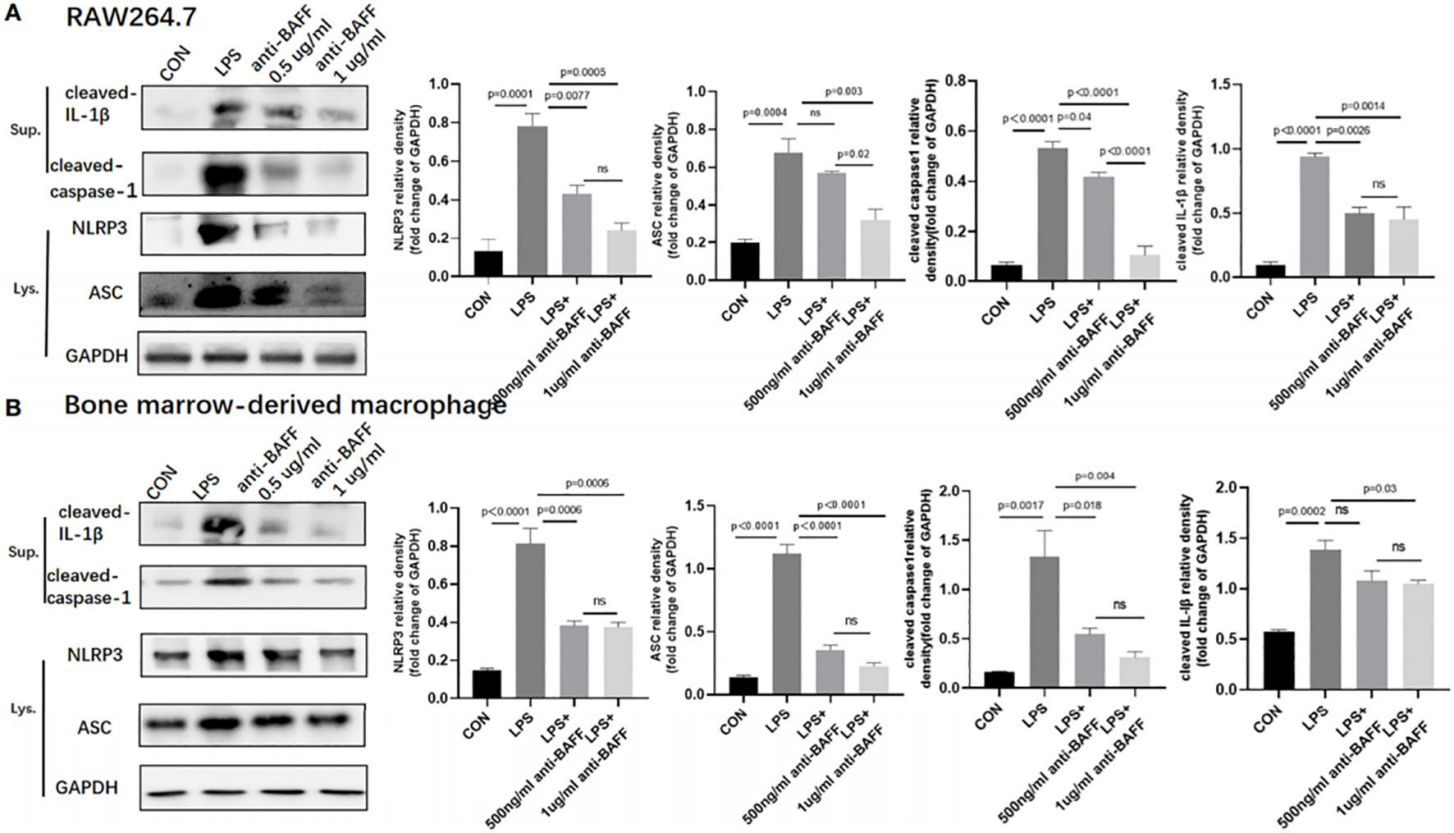
From Zhang, Ying et al. “BAFF Blockade Attenuates DSS-Induced Chronic Colitis via Inhibiting NLRP3 Inflammasome and NF-кB Activation.” Frontiers in immunology vol. 13 783254. 7 Mar. 2022.

In Zhang’s report, the murine cell line RAW264.7 was used *in vitro* experimental analysis. The authors reported that BAFF blockade significantly reduced ASC by western blot in LPS-induced RAW264.7 cells (**Figure 8A**). It is widely acknowledged that the RAW264.7 murine cell line lacks the expression of ASC, which can be attributed to epigenetic silencing, particularly DNA methylation (2–7). Therefore, the detection of ASC protein expression in ASC-deficient RAW264.7 cells in the present study made us confused.

**Figure 1:**
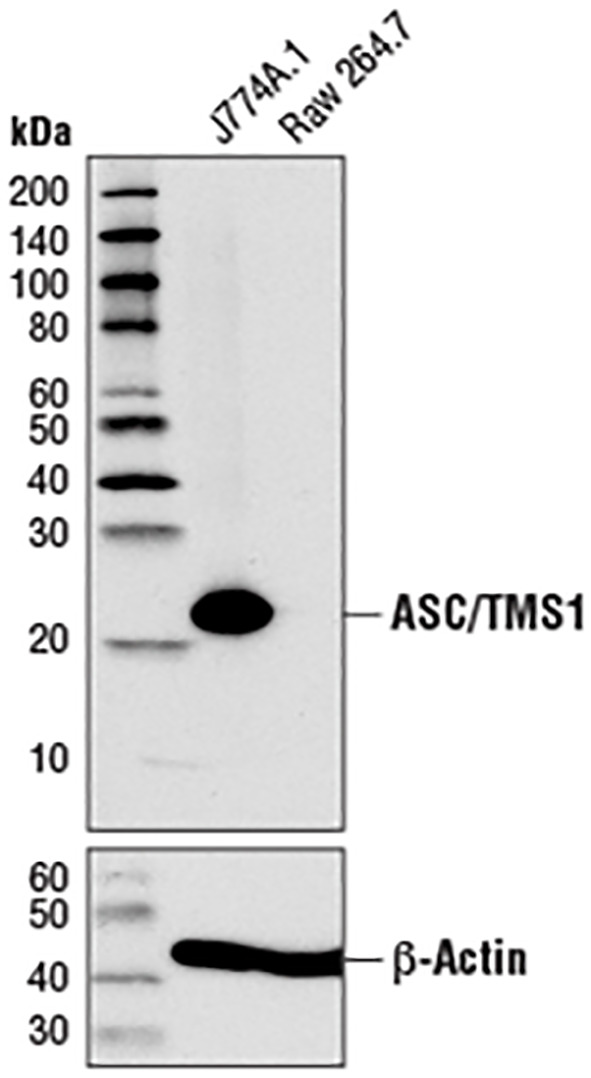
Western blot analysis of extracts from J774A.1 and RAW264.7 cells using ASC (D2W8U) rabbit monoclonal antibody (#67824) and β-actin (D6A8) rabbit monoclonal antibody (#8457). The data were downloaded from the website of Cell Signaling Technology company.

A further apparent problem is that in the section of MATERIALS & METHODS, the primary antibody anti-ASC, used in the western blot experiment, is produced by the Cell Signaling Technology company. As illustrated in **Figure 1**, the image from the Cell Signaling Technology website depicts a Western blot analysis of extracts from J774A.1 and Raw 264.7 cells utilizing ASC antibody (#67824, #37953). According to the instructions, ASC antibody (either #67824 or #37953) has explicitly stated that ASC protein in Raw264.7 cells cannot be detected (server as a negative control). Moreover, this ASC-deficient characteristic of RAW264.7 cells has been widely exploited in a multitude of studies as a cellular model to investigate ASC-independent inflammasome pathways or to examine the effects of ASC exogenous expression (4, 6, 8–12). For instance, Sun et al. demonstrated (5) that propofol treatment of RAW264.7 cells did not result in caspase-1 and gasdermin D cleavage. The exogenous expression of ASC in RAW264.7 cells was found to be a prerequisite for propofol-induced pyroptosis. To foster studies on the ASC adaptor, InvivoGen company has developed RAW-ASC cells (Cat. Code: raw-asc), which were generated by stable transfection of the murine ASC gene into the murine RAW 264.7 macrophage cell line, which is naturally ASC-deficient.

This discrepancy may be partially attributed to contamination of the samples and the use of incorrect reagents that cross-react with proteins unrelated to ASC in cellular extracts. An additional possibility is that the cultures examined may not contain the original RAW 264.7 cell line, potentially due to contamination with other cell types during the process of culturing and passaging. It may be necessary for the authors to confirm the identity of the RAW 264.7 cell line through the use of short tandem repeat analysis or other appropriate methods.

## 3 Conclusion

In conclusion, Zhang et al. have made a valuable contribution to our understanding of the role of BAFF in IBD pathogenesis. This work paves the way for new avenues of research and potential treatment strategies in inflammatory bowel diseases. Although the methodology and results of the study are praiseworthy, there is a need to reinforce the conclusions. It would be beneficial to address the discrepancy regarding ASC detection in RAW264.7 cells, which are known to lack ASC expression, in order to enhance the reliability of the *in vitro* findings. Furthermore, the validation of pivotal outcomes through the utilization of ASC-expressing RAW264.7 cell lines may facilitate the generation of more conclusive evidence regarding the impact of BAFF on the complete NLRP3 inflammasome.
